# Abortion after the Zika virus epidemic in Northeast Brazil

**DOI:** 10.11606/s1518-8787.2021055003053

**Published:** 2021-03-29

**Authors:** Debora Diniz, Marcelo Medeiros, Alberto Madeiro

**Affiliations:** I Universidade de Brasília Faculdade de Direito BrasíliaDF Brasil Universidade de Brasília. Faculdade de Direito. Brasília, DF, Brasil; II Princeton University Program of Latin American Studies PrincetonNew Jersey USA Princeton University. Program of Latin American Studies. Princeton. New Jersey, USA; III Universidade Estadual do Piauí Núcleo de Pesquisa e Extensão em Saúde da Mulher TeresinaPI Brasil Universidade Estadual do Piauí. Núcleo de Pesquisa e Extensão em Saúde da Mulher. Teresina, PI, Brasil

To the Editor: Northeast Brazil was the epicenter of a Zika virus outbreak between 2015 and 2016. During the epidemic, health officials have urged women to avoid pregnancy. Data from 2016 show that 66% of women from the Northeast avoided becoming pregnant during the epidemic^1^. However, most pregnancies in low socio-economic areas are unplanned^2^. Therefore, it is possible that not all women were able to prevent pregnancy and, instead, avoided the risk of congenital Zika syndrome by undergoing an unsafe abortion, since abortion is illegal in Brazil. To study the possible impact of the Zika outbreak on abortion in Northeast Brazil, we conducted a representative household survey of 1,008 women, combining interviewer-administered and self-administered questionnaires (anonymous, using the ballot-box technique).

The data showed there was a decline of abortions in 2019, when compared with similar surveys in 2010 and 2016. About 12% of women in 2019 declared having had at least one abortion in their lives, compared with 18% in 2017 and 20% in 2016. Despite the decline, our results show that abortion is frequent in the region. However, we did not identify any important differences in the abortion rates related to the Zika virus epidemic. Abortion rates in 2017–2018 were similar among women who had experienced (2.8%) or had not experienced (2.5%) the symptoms of Zika, dengue or chikungunya. In addition, we found no evidence that the higher prevalence of children with the congenital Zika syndrome born from low-educated women is the result of a lack of resources to have an abortion^3^. On the contrary, abortion rates are higher among these women. This suggests the higher prevalence of the syndrome may be a combination of fewer resources to avoid pregnancies and more exposure to environmental risks.

As the structural conditions for a new Zika outbreak remain unaltered, these results suggest that a strategy to control the congenital syndrome must go beyond official behavioral recommendations and include broad reproductive health measures, as well as access to contraceptives and to safe abortion.
